# Recent advances: role of mycolactone in the pathogenesis and monitoring of *Mycobacterium ulcerans* infection/Buruli ulcer disease

**DOI:** 10.1111/cmi.12547

**Published:** 2015-12-29

**Authors:** Fred Stephen Sarfo, Richard Phillips, Mark Wansbrough‐Jones, Rachel E. Simmonds

**Affiliations:** ^1^ Department of MedicineKwame Nkrumah University of Science & TechnologyKumasiGhana; ^2^ Division of Cellular and Molecular MedicineSt George's, University of LondonLondonUK; ^3^ School of Biosciences and MedicineUniversity of SurreyGuildfordUK

## Abstract

Infection of subcutaneous tissue with *Mycobacterium ulcerans* can lead to chronic skin ulceration known as Buruli ulcer. The pathogenesis of this neglected tropical disease is dependent on a lipid‐like toxin, mycolactone, which diffuses through tissue away from the infecting organisms. Since its identification in 1999, this molecule has been intensely studied to elucidate its cytotoxic and immunosuppressive properties. Two recent major advances identifying the underlying molecular targets for mycolactone have been described. First, it can target scaffolding proteins (such as Wiskott Aldrich Syndrome Protein), which control actin dynamics in adherent cells and therefore lead to detachment and cell death by anoikis. Second, it prevents the co‐translational translocation (and therefore production) of many proteins that pass through the endoplasmic reticulum for secretion or placement in cell membranes. These pleiotropic effects underpin the range of cell‐specific functional defects in immune and other cells that contact mycolactone during infection. The dose and duration of mycolactone exposure for these different cells explains tissue necrosis and the paucity of immune cells in the ulcers. This review discusses recent advances in the field, revisits older findings in this context and highlights current developments in structure‐function studies as well as methodology that make mycolactone a promising diagnostic biomarker.

## Introduction

*Mycobacterium ulcerans* (MU) infection can cause Buruli ulcer (BU), a chronic necrotizing skin infection with high prevalence in rural West Africa (Junghanss *et al*., 2014; O'Brien *et al*., 2014). It may manifest initially as a pre‐ulcerative nodule, plaque or a rapidly progressing oedematous lesion. Over a few weeks, these lesions break down to form characteristic ulcers with undermined edges, which are associated with extensive necrosis alongside minimal inflammatory response. In contrast to other pathogenic mycobacteria, which are facultative intracellular pathogens of macrophages, MU is seen as extracellular clusters of bacilli lying within areas of coagulative necrosis that extend some distance from the site of bacterial colonisation (Forbes *et al*., 1954). This observation led to early proposals that MU secretes an exotoxin (Connor *et al*., 1965; Connor *et al*., 1966). A major breakthrough occurred in 1999 with the purification and isolation of a cytotoxic molecule from the acetone soluble fraction of lipid extracts of MU. This lipid toxin, designated as mycolactone, was shown to be cytopathic to cultured L929 murine fibroblasts. Intradermal inoculation of purified toxin into guinea pigs produced a lesion histologically similar to BU with necrosis of subcutaneous fat. Furthermore, inoculation of an isogenic toxin‐negative mutant of MU caused a granulomatous lesion typical of the inflammatory response to other mycobacteria with phagocytosed MU visible within macrophages and none of the characteristic fat necrosis (George *et al*., 1999). Mycolactone is now known to be responsible for the immunosuppression and tissue necrosis in BU, as well as the painlessness of BU ulcers; one reason why patients often delay treatment (Goto *et al*., 2006).

**Figure 1:**
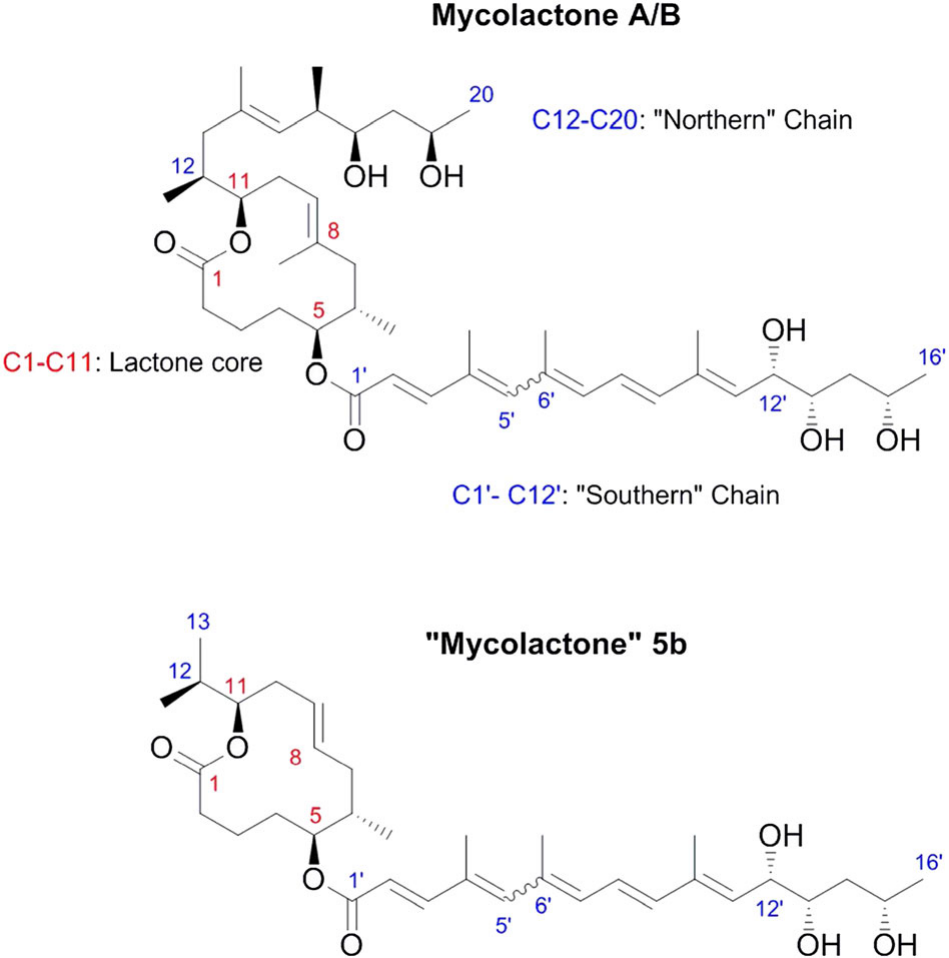
Molecular structure of mycolactone A/B. The chemical structure of mycolactone A/B has a core cyclic lactone ring (C1–C11) and two polyketide‐derived highly unsaturated acyl side chains. The upper ‘Northern’ chain consists of C12–C20 and the longer ‘Southern’ chain is numbered C1′–C16′. The numbering reflects the natural synthetic pathway of mycolactone by the polyketide synthase enzymes in MU. Under common laboratory conditions and light, mycolactone exists as spontaneously forming geometric isomers centered around the double bond at C4′C5′ (indicated by the wavy line between C5′ and C6′) in a 3:2 ratio. The structure of the variant mycolactone‐like molecule **5b** is also shown; this lacks the C8 methyl and the Northern chain.

Mycolactone is a polyketide composed of an invariant 12‐membered lactone ring to which two polyketide‐derived highly unsaturated acyl side chains are attached (Fig. 1). The upper ‘Northern’ chain is invariant, whereas variations in the ‘Southern’ chain give rise to different congeners of mycolactone. The initial molecule reported was identified on thin layer chromatography (TLC) as a light yellow, ultraviolet‐active lipid band with a retention factor value of 0.23 (George *et al*., 1999). Analysis of the toxin by mass spectrometry (MS) under microspray conditions showed a strong peak at m/z 765 with a formula of C_44_H_70_0_9_Na corresponding to the sodium adduct of mycolactone. Mycolactone synthesis depends on a 174 kb megaplasmid named pMUM001, which has three large genes (*mlsA1*, *mlsA2* and *mlsB*) encoding the modular type I polyketide synthases as well as accessory genes (Stinear *et al*., 2004). MlsA1 and MlsA2 form a nine‐extension module complex that synthesizes the mycolactone core, while the MlsB is a single polypeptide, comprising seven extension modules that are required for the synthesis of the side chain. Accessory proteins, such as mup045 and mup038, are thought to be required for joining of the subunits. However, these genes are necessary (but not sufficient) for mycolactone synthesis (Porter *et al*., 2009; Porter *et al*., 2013). Variants or congeners of mycolactone differ in the positions of hydroxyl groups in the Southern chain and exhibit differences in biological activity depending on the origin of strains of MU. Pathogenic human strains from Africa, Australia and China predominantly produce mycolactone A/B (m/z 765), C (m/z 749) and D (m/z 779), respectively, but each strain also produces minor quantities of the other two congeners (Mve‐Obiang *et al*., 2003). The most cytotoxic congener *in vitro* is mycolactone A/B. Notably, other genetically related organisms pathogenic to fish and frogs can also produce variants of mycolactone E (m/z 737) and F (m/z 723) (Mve‐Obiang *et al*., 2005; Ranger *et al*., 2006; Pidot *et al*., 2008).

### Kinetics of mycolactone production during Buruli ulcer pathogenesis and antibiotic treatment

Recent advances in methodology have allowed us, for the first time, to appreciate how mycolactone concentration changes in MU infections in experimental animal models and in human patients. Detection of mycolactone currently uses approaches that involve the extraction of acetone soluble lipids from tissue samples. Early experiments used labelled mycolactone, extracted from MU grown in C_14_ (Hong *et al*., 2008), but more recently, mycolactone concentration has been quantified by different methods. Mass spectroscopic (MS) detection of mycolactone employing high‐performance liquid chromatography with mass spectrometry (LC‐MS) to identify the parent ion (m/z) followed by MS–MS fragmentation to produce characteristic daughter ions (Hong *et al*., 2003) is a highly sensitive and specific approach. The intensities of parent to daughter ion ratios on mass spectrometry of serial dilutions of synthetic mycolactone standards are used to plot calibration curves for mycolactone quantification (Sarfo *et al*., 2013; Sarfo *et al*., 2014). The utility of this method is limited to highly sophisticated laboratories for research purposes. Others include assessing cytotoxic activity against L929 fibroblasts compared with a purified mycolactone standard. Because L929 fibroblasts are exquisitely sensitive to mycolactone, this approach can detect small amounts of mycolactone. The specificity of this approach is however limited by the fact that other cytotoxic components generated in infected tissue may also be present in these preparations (perhaps including mycolactone's breakdown products). Another experimental approach that is undergoing validation involves fluorescent TLC in which mycolactone A/B, but not most other acetone soluble lipids, fluoresce under UV light (Spangenberg *et al*., 2010). This method involves the estimation of band intensities relative to mycolactone standards and may be of utility in endemic countries as it can be performed without access to expensive and technically demanding equipment.

By means of these complementary approaches, mycolactone was shown to be present within human BU lesions (Sarfo *et al*., 2010). Cytotoxicity assays gave positive results for 92% of patients tested (mycolactone concentration estimates ranged from 0.3 to 6 µg/punch biopsy), whereas MS was able to detect mycolactone in 77% of the same patients (mycolactone concentration estimates ranged from 10 ng to 2 µg/punch biopsy) (Sarfo *et al*., 2014). Furthermore, mycolactone has also been detected in peripheral blood mononuclear cells (and nearly all other tissues apart from the brain) of mice after subcutaneous injection of 300 µg of C_14_‐labelled mycolactone (Hong *et al*., 2008) and in the sera of BU patients by MS (Sarfo *et al*., 2011), proving that mycolactone can escape the region of local MU infection.

One of the commonly used models of BU is a mouse model, in which inoculation of MU into footpads causes lesions to develop over several weeks. Mycolactone has now been detected in experimentally infected footpads by various methods. These studies agree that mycolactone is detectable by MS (Sarfo *et al*., 2013) or fluorescent TLC (Converse *et al*., 2014) within a few days of the onset of footpad swelling, which precedes ulceration in this model. At its peak, it is estimated that an infected footpad can contain 50–200 ng mycolactone (Sarfo *et al*., 2013; Converse *et al*., 2014). Antibiotic treatment is known to cause a decrease in footpad swelling along with reduced MU colony counts, and this is associated with decreased mycolactone concentration. Cytotoxicity assays revealed mycolactone‐like activity to be present earlier than whole mycolactone molecules (by MS), and this persisted even after viable bacilli had been eliminated (Sarfo *et al*., 2013).

Similarly, standard antibiotic therapy for BU (daily oral rifampicin and intramuscular streptomycin for 8 weeks) causes a substantial decrease in mycolactone detected in skin biopsies by both cytotoxicity and MS (Sarfo *et al*., 2014). However, in some cases, mycolactone could still be detected in culture‐negative samples suggesting that it may remain in tissue for a time after the organisms have been killed. This may explain the clinical observation of profound variations in the time to healing of similar‐sized BU lesions (Sarfo *et al*., 2014) because low doses of mycolactone are known to have wide reaching biological effects, as discussed in the following section. Alternatively, detection of mycolactone in these lesions may also imply that viable organisms are still present in some lesions despite negative cultures; culture is only positive in 40–60% of untreated BU lesions (O'Brien *et al*., 2014), whereas mycolactone is detectable in 77–92%. This discrepancy has implications for the duration of antibiotic therapy, currently recommended to be 8 weeks. Mycolactone is therefore a potential tool for monitoring the response of individuals with delayed healing.

## Molecular targets of mycolactone

Recently, two direct molecular targets of mycolactone have been described. Because both of these targets are widely expressed, these biological effects of mycolactone should underpin the previous findings on cellular phenotype that will be considered in this review. Furthermore, a specific target of mycolactone was recently reported to explain the painlessness of BU lesions (Marion *et al*., 2014) Mycolactone was reported to dose‐dependently induce hyperpolarisation of murine neurons and to inhibit the binding of angiontensin II to the type 2 angiotensin II receptor (AT_2_R) with an IC_50_ of 3 µg ml^−1^. While AT_2_R was required for this effect, the hyperpolarisation itself was found to be mediated by AT_2_R‐dependent activation of phospholipase A2, the release of arachidonic acid by COX‐1 and activation of the K^+^ channel‐TRAAK by prostaglandin E2 (Marion *et al*., 2014). It will be important to investigate this mechanism in BU patients, because the function (and indeed expression) of AT_2_R in healthy adult humans outside of the brain is controversial (Ichiki, 2013).

### Wiskott–Aldrich syndrome protein hyperactivation

**Figure 2:**
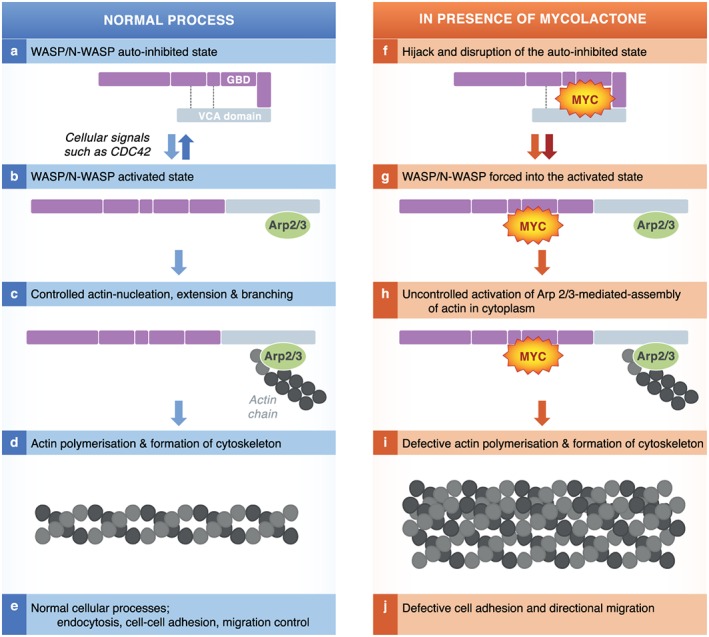
Mycolactone causes hyperactivation of WASP. WASP and N‐WASP are modular scaffolding proteins that exist in auto‐inhibited conformation in which the VCA (verprolin‐cofilin‐acidic) domain (yellow) is occluded by an intramolecular interaction (dotted line; a). Activation of WASP and N‐WASP occurs by disruption of the intermolecular interaction by a variety of ligands including the cell cycle regulator CDC42 and binding/activation of the Arp2/3 complex (b). The VCA domain of WASP, Arp2/3 complex and G‐actin forms a nucleating centre for incorporation of actin subunits for growth of actin filaments (c, d, e). Actin polymerisation and formation of cytoskeleton is crucial for endocytosis, cell‐to‐cell adhesion and migration of cells. Mycolactone hijacks and disrupts the auto‐inhibited state of WASP/N‐WASP (f). This forces WASP into the activated state with Arp2/3 bound (g). Mycolactone causes increases in the rate of Arp2/3‐mediated actin assembly, outside of normal cellular control (h) Unregulated actin polymerisation leads to defective cytoskeleton formation and loss cell adhesion and apoptosis (i, j).

One of the long‐known consequences of mycolactone exposure on cells cultured *in vitro* is the cytoskeletal rearrangement and detachment of cells from the culture dish. Recently, this was reported to be because of alterations in actin dynamics and the direct binding of mycolactone to Wiskott–Aldrich syndrome protein (WASP) and neural WASP (N‐WASP) (Guenin‐Mace *et al*., 2013). These are members of a family of scaffold proteins transducing a variety of signals into dynamic remodelling of the actin cytoskeleton via interaction of their C‐terminal verprolin‐cofilin‐acidic (VCA) domain with the ARP2/3 actin‐nucleating complex [reviewed by Thrasher *et al*. (2010)]. Using a combination of biochemical assays, cellular imaging and animal models, it was reported that mycolactone can hijack these actin‐nucleating factors (Fig. 2). By disrupting WASP autoinhibition, mycolactone can lead to uncontrolled activation of ARP2/3‐mediated assembly of actin in the cytoplasm. In HeLa cells, this then leads to concentration of ARP2/3 in the perinuclear region, resulting in defective cell adhesion and directional migration. Reductions in the re‐adhesion of dissociated, mycolactone‐treated, HeLa cells could be restored by co‐administration of the N‐WASP inhibitor wiskostatin, supporting this.

### Inhibition of co‐translational translocation via Sec61

**Figure 3:**
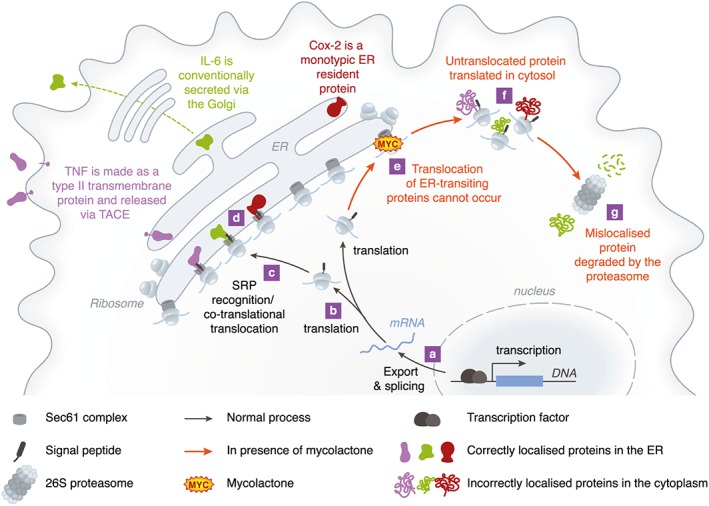
Mycolactone inhibits the co‐translational translocation of proteins via Sec61. Sec61‐dependent, ER‐transiting proteins are derived from mRNAs (a) and usually have a signal peptide sequence at the amino terminus (b). Once this is formed, translation pauses and the signal peptide is recognized by the SRP (not shown), which transports the ribosome/mRNA/nascent peptide complex to the Sec61 complex at the ER membrane (c). The hydrophobic signal peptide interacts with Sec61 and translation continues, directly into the ER lumen (d); a process further facilitated by chaperones such as BiP (not shown). A similar process occurs for transmembrane proteins (TNF), monotypic proteins (COX‐2) and conventionally secreted proteins (IL‐6). In the presence of mycolactone, translocation cannot occur (e) so translation takes place in the cytoplasm instead (f), and the proteins, recognized by the cell as being in the wrong compartment, are destroyed almost immediately by the 26S proteasome (g). This means that induced proteins can never be detected, and constitutive proteins are lost from the cell as they cannot be replaced during normal protein recycling.

In addition to cytopathic effects, another well‐established activity of mycolactone is the inhibition of cytokine production. Recently, the molecular mechanism underlying this was also reported, as mycolactone profoundly inhibits the function of the Sec61 translocon. This essential ‘gate keeper’ is responsible for protein translocation into the endoplasmic reticulum (ER), something that must occur for about 30–50% of proteins made by mammalian cells, not only cytokines but also membrane receptors and proteins that function inside the ER itself. Normally, most ER‐transiting proteins undergo ‘co‐translational translocation’, i.e. the ribosome becomes attached to the ER membrane via Sec61 accessory proteins and the signal peptide, and the translating peptide is synthesized directly into the ER lumen for folding by chaperones [reviewed by Zimmermann *et al*. (2011)]. Using detailed biochemical assays, on a range of different proteins, it was shown that the presence of mycolactone prevents this from taking place, and instead the proteins are translated in the cytosol where they are marked for rapid destruction by the proteasome (Hall *et al*., 2014) (Fig. 3). Formal evidence clearly supported translocation blockade affecting IL‐6, prepro‐α factor and β lactamase (conventionally secreted proteins; a cytokine and two non‐mammalian model proteins used routinely in translocation assays), COX‐2 (an ER‐resident enzyme), tumour necrosis factor and thrombomodulin (both type I membrane proteins). Furthermore, cessation of production of N‐linked glycosylated proteins was demonstrated by metabolic labelling, indicating that potentially hundreds of proteins are influenced by this mechanism in every cell. Therefore, while the purpose of this study was to explain the lack of inflammation in BU, the identified mechanism probably impacts the pathogenesis in multiple ways (Hall *et al*., 2014; Ogbechi *et al*., 2015).

## Cellular consequences of mycolactone exposure

The biological function of mycolactone A/B has been intensively studied *in vitro* and *in vivo* by using purified lipid extracts from cultured MU, chemically synthesized mycolactone, or by comparing the effects of wild‐type MU with those of mycolactone‐negative mutant strains with broadly comparable results. Mycolactone seems to diffuse passively across the cell membrane because uptake of fluorescently labelled mycolactone by fibroblasts was both non‐saturable and non‐competitive with excess mycolactone (Snyder *et al*., 2003). Bodipy‐mycolactone appears to be localized in the cytosol but is excluded from the nucleus (Chany *et al*., 2011). At least some of the effects of mycolactone have also been shown to be irreversible; for example, suppression of cytokine production still occurs 24–48 h after mycolactone is removed from cell culture media (Hall *et al*., 2014). In the following review of the cellular actions of mycolactone, we have focused on the time of exposure and dose of mycolactone and revisited some of the findings in the light of our new knowledge about the molecular mechanisms.

### Immune cells

Much research has been directed towards the effects of mycolactone on immune cells, in order to better understand the mechanism of immune suppression, which affects both innate and adaptive immune responses. For instance, in mycolactone‐negative strains of MU (Adusumilli *et al*., 2005), and during antibiotic treatment of BU patients (Schutte *et al*., 2007), infection is associated with granulomas that are initiated by infected macrophages. However, in active BU, these are rarely observed, presumably because of the action of mycolactone.

#### Monocytes and macrophages

Macrophages (and their circulating precursors, monocytes) are vital components of innate and anti‐mycobacterial immunity. Despite the fact that mycolactone has been shown to interfere with phagocytosis by J774 macrophages (Adusumilli *et al*., 2005), a transient intra‐macrophage growth phase has been shown for MU (Torrado *et al*., 2007). Once internalized, the toxin also modulates macrophage microbicidal activity triggered by IFN‐γ via inhibition of phagosome maturation/acidification and nitric oxide production with consequent increased intra‐macrophage bacterial load (Torrado *et al*., 2010). Mycolactone‐negative strains of MU survive better inside J774 macrophages (Adusumilli *et al*., 2005), and wild‐type infected macrophages eventually succumb to mycolactone's cytotoxic effects leading to the predominant finding of MU in tissue as extracellular clusters of bacilli. Macrophages are sensitive to mycolactone's cytotoxic effect *in vitro* but at relatively high doses and after prolonged exposure (several days) (Simmonds *et al*., 2009; Hall *et al*., 2014). Notably, during antibiotic treatment of BU patients, MU can once again be detected within macrophages (Schutte *et al*., 2009).

Mycolactone also affects the function of monocytes/macrophages independently of contact with MU. Purified mycolactone dose‐dependently inhibits production of cytokines, chemokines, other secreted immune modulators (Table 1) and intracellular effector molecules such as COX‐2 (Simmonds *et al*., 2009; Hall *et al*., 2014) at doses of 50–125 ng ml^−1^ mycolactone. While IL‐1β was also suppressed by mycolactone, this was less efficient than for the other, conventionally secreted, cytokines (Simmonds *et al*., 2009). In the context of our improved understanding of mycolactone's inhibition of Sec61 translocation, it is interesting to note that IL‐1β does not undergo this process and is instead released from a cytoplasmic pro‐IL‐1β precursor by caspases activated via the inflammasome (Rathinam *et al*., 2012).

**Table 1:** Inhibition of cytokine, chemokine and other immune modulators by mycolactone.

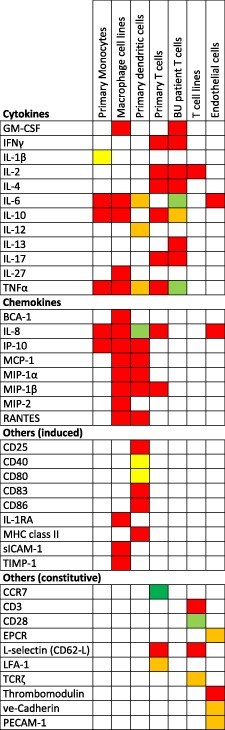

Summary of findings of multiple publications that have reported the effect of mycolactone directly on the production of various cytokines, chemokines and other proteins by monocytes (Simmonds *et al*., 2009), macrophages (Hall *et al*., 2014), dendritic cells (Coutanceau *et al*., 2007), primary T‐cells (Phillips *et al*., 2009, Guenin‐Mace et al. 2011), T‐cell lines (Pahlevan *et al*., 1999, Boulkroun *et al*., 2010) and endothelial cells (Ogbechi *et al*., 2015). Colour coding is as follows: Red, profound inhibition; orange, some inhibition; yellow, little inhibition; green, no inhibition; dark green, increased expression; white, not studied

Notably, in the process of uncovering the protein translocation defect caused by mycolactone that explains the loss of these proteins (see discussions earlier), the transcription or export (Simmonds *et al*., 2009) and translation (Hall *et al*., 2014) of these proteins were not directly affected. Mycolactone had no effect on the TLR‐dependent activation of the MAPK and NF‐κB signalling pathways, ribosomal association was normal and there was no evidence of conventional cellular stress during short exposure times (<5 h). Recently, hyperpolarisation of RAW264.7 macrophages was found to occur rapidly (within 20 mins) of high dose (5 µg ml^−1^) mycolactone exposure, but not at lower doses (Marion *et al*., 2014).

#### Dendritic cells

Both immature and mature primary human dendritic cells are sensitive to mycolactone‐induced apoptosis at concentrations above 50 ng ml^−1^. Below this concentration, mycolactone strongly affected the maturation of dendritic cells, inhibited the induction of co‐stimulatory molecules, limited their migratory properties but not their phagocytic activity and exerted an apparently selective effect on the secretion of inducible chemokines by dendritic cells in response to Toll‐like receptor ligands (Coutanceau *et al*., 2007). In particular, mycolactone blocked the production of chemokines, whereas the expression of inflammatory cytokines was less affected at the doses used (Table 1). As key players in the initiation of adaptive immune responses, mycolactone‐dependent suppression of co‐stimulatory molecules, chemokines and cytokines would severely inhibit the capacity of dendritic cells to prime cellular immune responses. The loss of co‐stimulatory molecules, cytokines and chemokines likely results from blockade of Sec61 translocation in these cells.

#### T‐cells

Experiments using a crude preparation of mycolactone were the first to suggest that the immunosuppressive properties of mycolactone extend to lymphocytes because it inhibits production of IL‐2 by activated T‐cell lines (Pahlevan *et al*., 1999), a finding later confirmed (Phillips *et al*., 2009; Boulkroun *et al*., 2010). Mycolactone also profoundly inhibits the phorbol myristate acetate (PMA)/ionophore‐induced production of many cytokines and chemokines by primary human CD4^+^ T lymphocytes. Antigen‐dependent production of many Th‐1, Th‐2 and Th‐17 cytokines by T‐cells from BU patients was also diminished (Phillips *et al*., 2009) (Table 1). Defective production of IFN‐γ in MU antigen stimulated peripheral blood cells from patients with active BU disease has been reported using various assays (T‐cell anergy) (Gooding *et al*., 2003; Prevot *et al*., 2004; Westenbrink *et al*., 2005; Phillips *et al*., 2006), providing further evidence of a lack of co‐stimulation by dendritic cells. This resolves after either surgical excision of ulcers (Yeboah‐Manu *et al*., 2006) or after curative antibiotic therapy (Schutte *et al*., 2007; Sarfo *et al*., 2009) supporting the involvement of mycolactone in these defects. Notably, primary T‐cells are relatively resistant to the cytotoxic effects of mycolactone at concentrations of up to 1 µg ml^−1^ (Boulkroun *et al*., 2010; Guenin‐Mace *et al*., 2011).

Mycolactone also has profound effects on the crucial process of T‐cell homing *in vivo*, because subcutaneous administration of large doses (50 or 100 µg) of mycolactone leads to massive depletion of T‐cells in peripheral lymph nodes (PLNs) (Guenin‐Mace *et al*., 2011). This was associated with defective expression of the cell adhesion/homing receptor L‐selectin (CD62‐L). Pre‐exposure of T‐cells to 100 ng ml^−1^ mycolactone for 16 h severely impaired the capacity of T‐cells to reach peripheral lymph nodes after adoptive transfer, respond to chemotactic stimuli and expand upon antigenic stimulation *in vivo*.

With hindsight, it now seems likely that the expression defects for these ER‐transiting proteins may be mediated by translocation blockade. It also helps explain some of the contradictory findings of the previous studies. For instance, when investigating the mechanisms by which mycolactone modulates IL‐2 production after antigenic stimulation in Jurkat T‐cells, identified mechanisms included reductions in reporter gene activity for NF‐κB, AP‐1, NFAT/AP‐1 and the IL‐2 promoter. Conversely, despite ionomycin/PMA activation‐induced IL‐2 production being strongly suppressed by mycolactone, this was associated with increased activation of AP‐1 and the IL‐2 promoter (Boulkroun *et al*., 2010). Furthermore, mycolactone was found to reduce levels of L‐selectin, without affecting its key transcription factor KLF‐2. Here, mycolactone also reduced the expression of microRNA let‐7b, although over‐expressing it could not restore the ability of the T‐cells to produce L‐selectin protein. These examples highlight the important point that translocation blockade effectively ‘trumps’ any effect of pre‐translocation events. Even if transcription or translation of a particular gene is increased or decreased by mycolactone; if the synthesized protein cannot be translocated into the ER, it cannot be produced by an affected cell.

Mycolactone exposure also has effects on the cytosolic component of T‐cells that are not confounded by translocation blockade. Mycolactone rapidly (within 30 min) alters the expression of several microRNAs including let‐7b (Guenin‐Mace *et al*., 2011). Let‐7b‐mediated fine‐tuning of many unidentified target genes would be expected to lead to changes in cellular phenotype. For instance, caspase‐3 was recently described as a target of let‐7b in mesenchymal stem cells (Ham *et al*., 2015), suggesting that reduced let‐7b may contribute to apoptosis in T‐cells. Furthermore, mycolactone exposure for 24 h led to hyperactivation of the Src‐family kinase, Lck, and was associated with depletion of intracellular calcium stores and down‐regulation of T‐cell receptor (CD3) expression (Boulkroun *et al*., 2010).

#### Neutrophils

Bacterial infection is normally associated with influx of neutrophils into infected tissue. This is notably absent in the necrotic centre of BU lesions, but it is seen in surrounding tissue (Hayman *et al*., 1985; Hayman, 1993). Neutrophil chemotaxis towards MU is poor compared with *M*y*cobacterium marinum*, but the defect is probably not because of mycolactone as the same also applied to a mycolactone‐negative mutant strain (Adusumilli *et al*., 2005). Neutrophils are naturally short‐lived, but mycolactone‐dependent toxicity was evident within 4 h at very high doses (5.3 µg ml^−1^) and was enhanced at lower doses (1 µg ml^−1^) after 24 h.

### Skin‐resident non‐immune cells

A diverse range of different cell types are found within skin tissue. These include fibroblasts, adipocytes, endothelial cells, keratinocytes, neurons and muscle cells. The effect of mycolactone on each of these cell types has now been studied.

#### Fibroblasts

Dermal fibroblasts are one of the main constituents of skin dermis, and while no study has examined the effect of mycolactone on primary human skin fibroblasts, L929 mouse fibroblasts are the most mycolactone‐sensitive cell identified to date. Serial dilutions of mycolactone down to 0.025 ng ml^−1^ led to a cytopathic effect with cytoskeletal rearrangement at 12 h, rounding up of cells within 24 h and detachment from the plate by 48 h (George *et al*., 1999). We can now consider that this may be caused by the hyperactivation of WASP. The changes were associated with cell cycle arrest in the G_0_/G_1_ phase, and after 3–5 days, L929 cells undergo apoptotic cell death at mycolactone concentrations as low as 0.002 ng ml^−1^ (George *et al*., 2000). At higher concentrations (15 µg ml^−1^), mycolactone causes rapid changes in cell permeability leading to cell death by primary necrosis (Adusumilli *et al*., 2005).

#### Adipocytes

Adipocytes form the fatty layer of the subcutis within which MU is often found (Junghanss *et al*., 2014). Histological examination of MU‐infected guinea pig skin (George *et al*., 2000) and human skin (Walsh *et al*., 2005) shows evidence of adipose cell death by apoptosis.

To date, the effect of mycolactone has only been investigated on the human cell line SW872, derived from a liposarcoma (Dobos *et al*., 2001). It has recently been established that these cells express markers consistent with preadipocytes, but no adipocyte markers including FABP4 or the presence of lipid droplets (Nativel *et al*., 2013). Nevertheless, these cells seem relatively resistant to prolonged exposure to 1 µg ml^−1^ mycolactone, with even 5 days exposure leading to only low levels of apoptosis and higher levels of necrosis (Dobos *et al*., 2001). In these cells, cellular cytotoxicity ascribed to MU was rather suggested to be becuase of other components of the filtrate such as lipoprotein complexes.

#### Endothelial cells

Endothelial cells pervade the skin tissue, both in the microvasculature and lining the larger arteries and veins. They play important roles in the immune response and also a key role in the maintenance of intravascular fluidity by regulating blood coagulation. Recently, it was demonstrated that primary human dermal microvascular endothelial cells are highly sensitive to as little as 7 ng ml^−1^ mycolactone (Ogbechi *et al*., 2015). These cells died by apoptosis after 3–4 days exposure, but within 24 h, it caused the depletion of the anticoagulant receptor thrombomodulin from the surface of the cells. As a result, mycolactone‐exposed cells lose their crucial ability to activate protein C, a key anticoagulant protein. Other cell surface molecules (PECAM‐1, ve‐Cadherin and the endothelial cell protein C receptor) were more resistant to mycolactone but were still reduced. Loss of thrombomodulin was shown to be via Sec61 translocation blockade, and because this is a constitutively expressed protein, it was lost steadily from the cells at the known turnover rate. Notably, thrombomodulin was also reduced in the endothelial cells of BU patient biopsies and was associated with fibrin deposition in the tissue.

#### Keratinocytes

Keratinocytes display an unusual phenotype in BU. While most cells become necrotic, the epidermis becomes hyperplastic and psoriatic (Junghanss *et al*., 2014). These cells have a central role in wound healing and repair, forming a protective epithelial barrier over the wound bed. Thrombomodulin expression by keratinocytes in the epidermis of some BU patients was found to be decreased (Ogbechi *et al*., 2015), although it is not yet clear what the relevance of this is.

In purified systems, mycolactone causes rounding up of primary human keratinocytes after 24 h exposure to >30 ng ml^−1^ mycolactone (Gronberg *et al*., 2010), although most cells were still alive at this time. Once again, this may result from WASP‐dependent mechanisms. Cell death occurred between 48 and 72 h after exposure to 100 ng ml^−1^ and above, although sub‐cytotoxic doses seemed to actually increase the proliferation of keratinocytes slightly. Cytotoxic doses (300 µg ml^−1^) were associated with rapid generation of reactive oxygen species (ROS) within 45 mins of exposure that could be completely prevented by the ROS inhibiting substance deferoxamine. This Fe^2+^ chelator could efficiently block mycolactone's cytotoxic effects as assessed by neutral red uptake (an indicator of lysosome numbers) but was less efficient at restoring metabolic activity as assessed by the vital stain WST‐1 (Gronberg *et al*., 2010).

#### Neurons

Loss of pain sensation in BU lesions has long been known and can also be detected during experimental infection with MU in the mouse footpad model using nociceptive reflex tests utilising von Frey filaments and foot retraction (Goto *et al*., 2006) or thermal stimuli and tail‐flick test (Marion *et al*., 2014). Both found a reduced perception of pain in swollen/oedematous infected footpads around 5–7 weeks after infection (Goto *et al*., 2006; Marion *et al*., 2014), but in longer infections that resulted in greater erosion of the footpad and ulceration pain reception appeared to return (Goto *et al*., 2006). Hypoesthesia has been reported to be dependent on the expression of AT_2_R, because genetic knockout of this gene resulted in restoration of normal latency period for sensing of pain from 11 to 8 s (Marion *et al*., 2014).

There is disagreement over whether nerves are degenerated in these models. Early work showed that MU invaded the nerves in the perineurium and extended to the endoneurium with vacuolar degeneration of myelin‐forming Schwann cells and some swollen nerve bundles massively invaded by acid‐fast bacilli (Goto *et al*., 2006). Furthermore, injection of 100 µg purified mycolactone also induced nerve damage; nerve bundles showed intraneural haemorrhage, neutrophilic infiltration and loss of Schwann cell nuclei with ultrastructural evidence of vacuolar change of myelin (En *et al*., 2008). These studies implicated mycolactone in the direct destruction of nerves; however, in a more recent study, mycolactone induced analgesia without ultrastructural evidence of neural degeneration (Marion *et al*., 2014). Indeed, in tissue culture, primary murine hippocampal neurons seem remarkably resistant to mycolactone's cytopathic effect, only displaying significantly increased 24 h cell mortality in 20 µg ml^−1^ mycolactone (Marion *et al*., 2014).

## Structure‐function studies of mycolactone

Because base hydrolysis of mycolactone (separating the lactone core and Northern chain) was shown to result in a molecule with ~1000‐fold reduced cytopathic activity towards L929 fibroblasts (Mve‐Obiang *et al*., 2003), it has been clear that the virulence of the molecule requires the Southern polyketide side chain. Recent advances in chemical synthetic approaches have facilitated the production of many variants of mycolactone providing more detailed insight into the relationship between structure and function (Table 2). These have examined not only the cytopathic effect against L929 fibroblasts (although this is the most widely used assay) but also direct binding to WASP and immunosuppressive activity (suppression of IL‐2 production by Jurkat T‐cells). Discussion of these findings uses the common numbering system for the carbons in the ‘wild‐type’ mycolactone A/B molecule (Fig. 1). Furthermore, because these studies by definition compare compounds with different molecular weights, they are more commonly reported using molar concentrations. For reference, a 200 ng ml^−1^ preparation of mycolactone A/B (sufficient to elicit a functional effect on most cells) is equivalent to 0.27 μM.

**Table 2:** Structure‐function studies of mycolactone variants.

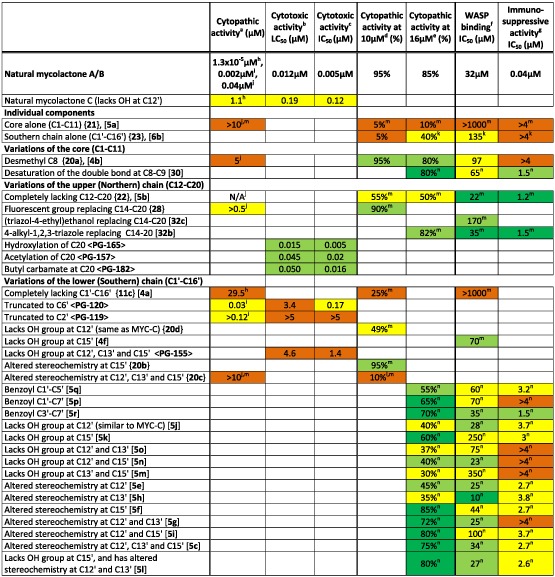

The lowest concentration of mycolactone inducing cell rounding at 24 h or 48 h.

The LC_50_ for induction of apoptosis by flow cytometry using annexin V/PI staining at 48 h (Scherr *et al*., 2013).

The IC_50_ for loss of metabolic activity by quantification of Alamar Blue staining at 48 h (Scherr *et al*., 2013).

The approximate proportion of cells rounded up at 48 h after treatment with the compound at a concentration of 10 μM (Chany *et al*., 2011).

The approximate proportion of cells that are permeable to trypan blue at 48 h after treatment with the compound at a concentration of 16 μM (Guenin‐Mace *et al*., 2015).

Approximate affinity of binding to WASP *in vitro* by competitive ELISA (Chany *et al*., 2014).

Approximate ability to prevent the production of IL‐2 by PMA/ionomycin activated Jurkat T cells (Guenin‐Mace *et al*., 2015).

Mve‐Obiang *et al*. (2003).

Scherr *et al*. (2013).

Chany *et al*. (2011).

Has a cyclohexyl ester in place of the core.

Did induce 100% rounding at 50 μM.

Different baseline molecules: Simplified core (desmethyl C8) so activity of variants compared with {**20a**} or [**4b**].

Different baseline molecules: Simplified core (desmethyl C8) and lacking the Northern chain so activity of variants compared with [**5b**].

Compound numbers are given in bold: <>, (Scherr *et al*., 2013); {}, (Chany *et al*., 2011); [], (Chany *et al*., 2014, Guenin‐Mace *et al*., 2015).

Mycolactone A/B has a m.w. of 743; therefore, 1 μM = 743 ng ml^−1^, 10 μM ≈ 7.4 µg ml^−1^ and 16 μM ≈ 12 µg ml^−1^. Other congeners of mycolactone have, where necessary, been converted from ng ml^−1^ values reported in the original reference according to their molecular weight. Colour coding is as follows: Red, profound inhibition; orange, some inhibition; yellow, little inhibition; green, no inhibition; dark green, increased expression; white; activity of mycolactone A/B.

One complication in understanding these data result from the fact that, in order to greatly simplify their synthesis, there is a whole class of mycolactone‐like molecules that lack the methyl group at C8 in the lactone core (so‐called C8‐desmethyl mycolactone) (Chany *et al*., 2011; Chany *et al*., 2014; Guenin‐Mace *et al*., 2015). Compared with the ‘wild‐type’ molecule, this has reduced activity (the lowest dose with cytopathic activity is 5 μM and the IC_50_ for IL‐2 is >4 μM vs. 40 nM for the ‘wild‐type’ molecule in both cases) and binds less well to WASP (IC_50_ 97 μM vs. 32 μM). However, tantalisingly, this activity can be restored by removing the Northern chain (IC_50_ for IL‐2; 1.2 μM, IC_50_ for WASP; 22 μM). In some of the investigations of structure/function, this molecule (named **5b**; Fig. 1) is the base‐line molecule that has been altered to investigate the effect of changes to the Southern chain (Guenin‐Mace *et al*., 2015).

Taken together, these studies clearly demonstrate that the lactone core alone has no discernible biological activity (Chany *et al*., 2011; Chany *et al*., 2014). Furthermore, variations in the Northern chain are generally well‐tolerated, such as the addition of different functional groups (including fluorescent tags and biotin) at C14 or C20 (Chany *et al*., 2011; Scherr *et al*., 2013; Chany *et al*., 2014; Guenin‐Mace *et al*., 2015). On the other hand, the Southern chain is much more sensitive to even small perturbations in structure. Similar to chemical modification of natural mycolactone A/B, truncation of the Southern chain to C6′ or C2′ (Scherr *et al*., 2013), or completely removing it (Chany *et al*., 2011) results in a molecule with much reduced cytopathic activity. The pattern and stereochemistry of the hydroxyl groups on the Southern chain is also important. While this had already been inferred by differences in the biological activity of naturally occurring conjoiners, these detailed studies have taken this much further. Nearly any change to the Southern chain reduces the molecule's immunosuppressive activity. Molecules completely lacking hydroxylation at C12′, C13′ and C15′ have lost most cytopathic and immunosuppressive activity (Chany *et al*., 2011; Scherr *et al*., 2013). Altering the stereochemistry of C12′ and C13′ also tend to have the effect of reducing the molecule's activity, depending on the precise context of the change, but alteration at C15′ seems to have little effect (Guenin‐Mace *et al*., 2015). The functional importance of the Southern chain is highlighted by the biological activity of this polyketide without the core or Northern chain. This molecule has near‐normal affinity for WASP (Chany *et al*., 2014) and retains some cytopathic activity when used at 16 μM (Guenin‐Mace *et al*., 2015).

The mycolactone‐like molecule **5b** (C8 desmethyl and lacking the Northern chain) is of particular interest because it retains the ability to suppress inflammation while having significantly reduced cytopathic activity. The lowest dose inducing a cytopathic effect in L929 fibroblasts has not been reported, but at doses of 10 μM and 16 μM, it has ~50% cytopathicity (Chany *et al*., 2011; Guenin‐Mace *et al*., 2015) and considerably higher concentrations (16 μM vs. 26 nM) were required to have a similar impact on the attachment/spreading of cells on a tissue culture surface (Chany *et al*., 2014). Further modification of this molecule by altering the stereochemistry, or constraining the molecule by the addition of aromatic rings within the polyketide chain, could not further enhance this desirable phenotype (Guenin‐Mace *et al*., 2015). However, **5b** is well‐tolerated following intraperitoneal injection into mice where it shows highly promising anti‐inflammatory effects in models of chronic skin inflammation and rheumatoid arthritis. It may therefore have therapeutic potential in the future as a potential immunosuppressant in diseases outside the realm of BU.

## Conclusion

The mycolactones are a unique and fascinating class of molecules. Not only are they central to the understanding of BU pathogenesis, they are also potentially important in the diagnosis and monitoring of BU. In the wider context, they are also helping us understand fundamental aspects of cell biology; mycolactone is one of only a handful of molecules that are capable of blocking translocation. However, mycolactones present several challenges in molecular investigations; not least obtaining the chemically pure material because mycolactone production in the laboratory is arduous and has several unstable intermediates. Synthetic mycolactone is preferable over compounds purified from MU becaue difficulties remain in the purification process, and MU itself is a slow‐growing organism requiring high‐level containment for culture. Furthermore, the physical characteristics of this lipid‐like molecule lend it an inherent ‘stickiness’, which can complicate analysis. This, together with its low antigenicity and immunosuppressive activity have hampered generation of mycolactone‐specific antibodies that could demonstrate the location and concentration of mycolactone within BU lesions and be used in biochemical assays. Novel approaches under development include synthesis of recombinant antibodies against mycolactone and mycolactone specific complexes using phage and yeast display methods (Naranjo *et al.*, unpublished observations). Such resources are expected to be of great utility in the future.

Despite these challenges, the past 10 years have yielded significant advances in our understanding of the biological activity of mycolactone. Central to this progress is the proposition of molecular mechanisms underpinning its cytotoxic and immunosuppressive properties. We eagerly await ratification of these findings by way of independent confirmation from different laboratories to demonstrate the commonality of the effects. Nevertheless, it is now clear that the interplay between mycolactone's cytotoxic and immunosuppressive effects varies depending on the cell the toxin is interacting with, the duration of exposure and the dose. These pleiotropic effects work together to create a complicated picture in the evolving lesion, whereby different functional effects may be apparent at the core of infection where the mycolactone concentration is highest, compared with the outer edge of biologically relevant mycolactone concentrations furthest from the clusters of MU. Intriguingly, this suggests that mycolactone may be operating via each or all of the described mechanisms in different cells at different times. This is especially true if you consider translocation blockade because of the large range of potentially affected proteins. Around 30–50% of protein coding genes have a signal peptide, but the response would be predicted to be dependent on the specific combination of such proteins expressed by a specific cell/condition and their sensitivity to mycolactone‐dependent blockade.

In the face of this complexity, the simple facts remain that mycolactone is the cause of the tissue necrosis, the impaired clearance of MU infection and the painlessness of the ulcers. The recent development of techniques for detection and quantification of mycolactone within host tissues has further deepened our understanding of the relationship between this toxic macrolide and clinical disease and has opened up a new vista for utilising mycolactone as a diagnostic and prognostic biomarker for the management of this neglected tropical disease.
